# Supplementary material for: Spirobifluorene-based polymers of intrinsic microporosity for the adsorption of methylene blue from wastewater: effect of surfactants
Source: R Soc Open Sci. 2020 Sep 9;7(9):200741. doi: 10.1098/rsos.200741 (PMC7540755; doi:10.1098/rsos.200741)
Supplement: Supporting Material [file rsos200741supp1.docx]

**Spirobifluorene-based polymers of intrinsic microporosity for the adsorption of methylene blue from wastewater: effect of surfactants**

**Entesar Al-Hetlani,^a^* Mohamed O. Amin,^a^ C. Grazia Bezzu,^b^ Mariolino Carta ^c^***

^a^ Department of Chemistry, Kuwait University, Faculty of Science, P.O. Box 5969, 13060 Safat, Kuwait. Email: [entesar.alhetlani@ku.edu.kw](mailto:entesar.alhetlani@ku.edu.kw)

^b^ School of Chemistry, Cardiff University, Cardiff CF10 3AT, U.K.

^c^ Department of Chemistry, Swansea University, College of Science, Grove Building, Singleton Park, Swansea, SA2 8PP, UK. Email: [mariolino.carta@swansea.ac.uk](mailto:mariolino.carta@swansea.ac.uk)

**Scheme S1:** Chemical structure of PIM-SBF-Me and PIM-SBF-tBu.

**Table S1:** Surface area, total pore volume and micropore volume of PIM-SBF- Me and PIM-SBF-tBu.

| **PIM** | **SA_BET_ (m^2^/g)** | **Total pore volume (mL/g)** | **Micropore volume (mL/g)** |
| --- | --- | --- | --- |
| PIM-SBF-Me | 752 | 0.54 | 0.28 |
| PIM-SBF-tBu | 882 | 0.70 | 0.31 |

**Figure S1:** The adsorption efficiency versus A) PIM-SBF-Me dosage B) PIM-SBF-tBu dosage.

**Figure S2:** Effect of initial MB concentration on adsorption efficiency of A) PIM-SBF-
Me and B) PIM-SBF-tBu.
